# Mallory-Weiss syndrome in four hemodialysis patients: a case study

**DOI:** 10.1186/s12882-023-03250-x

**Published:** 2023-06-27

**Authors:** Shuai-Shuai Shi, Xian-Zhu Yang, Xiao-ye Zhang, Lei Huang, Hui-Dan Guo, Shuang-fang Li, Wei Zhang, Yi-Qiang Zhang

**Affiliations:** 1 Department of Nephrology, Heji Hospital of Changzhi Medical College, Changzhi, 046011 Shanxi China; 2 Graduate School of Changzhi Medical College Changzhi, Shanxi, 046000 China; 3 Department of Endoscopy, Heji Hospital of Changzhi Medical College, Changzhi, 046011 Shanxi China; 4 Department of Radiology, Heji Hospital of Changzhi Medical College, Changzhi, 046011 Shanxi China; 5 Department of Biochemistry, Changzhi Medical College, 161 JieFang East Street, Changzhi, Shanxi 046000 P.R. China

**Keywords:** Mallory-Weiss syndrome, Hemodialysis, Treatment

## Abstract

**Background:**

Hemodialysis patients are prone to gastrointestinal bleeding, and Mallory-Weiss syndrome (MWS) is one of the causes. Mallory-Weiss syndrome is often induced by severe vomiting, manifests as upper gastrointestinal bleeding, and is self-limited with a good prognosis. However, mild vomiting in hemodialysis patients can lead to the occurrence of MWS, and the mild early symptoms are easy to misdiagnose, leading to the aggravation of the disease.

**Case presentation:**

In this paper, we report four hemodialysis patients with MWS. All patients displayed symptoms of upper gastrointestinal bleeding. The diagnosis of MWS was confirmed by gastroscopy. One patient had a history of severe vomiting; however, the other three reported histories of mild vomiting. Three patients received the conservative hemostasis treatment, and the gastrointestinal bleeding stopped. One patient underwent the gastroscopic and interventional hemostasis treatments. The conditions of three of the patients improved. Unfortunately, one of the patients died due to the cardia insufficiency.

**Conclusions:**

We think that the mild symptoms of MWS are easily covered up by other symptoms. This may lead to delays in diagnosis and treatment. For patients with severe symptoms, gastroscopic hemostasis is still the first choice, and interventional hemostasis can also be considered. For patients with mild symptoms, drug hemostasis is the first consideration.

## Background

Hemodialysis (HD) patients are prone to gastrointestinal bleeding, and one of the causes is Mallory-Weiss syndrome (MWS). MWS refers to non-transmural lacerations of the esophagogastric junction caused by severe vomiting and other inducements [1]. In most cases, the disease is self-limited and benign. However, HD patients can develop MWS after mild vomiting. The early symptoms of hematemesis are not typical, which leads to a delay in the diagnosis, and are easily complicated by serious gastrointestinal bleeding and even death. This study further explored the diagnosis and treatment of MWS in HD patients by reviewing and analyzing the clinical data, gastroscopic examination results, and follow-up data after treatment.

## Case presentation

This study describes four patients receiving maintenance HD who also had MWS. The general condition of the patients, their clinical presentation on admission, gastroscopy results, treatment histories, and prognoses are summarized below. The four patients are subsequently referred to as P1, P2, P3, and P4. The case data for each patient are as follows:

1. General patient condition

The four patients were receiving maintenance HD treatment at our hospital. The underlying disease of P1 and P2 was primary kidney disease, and the underlying disease of P3 and P4 was diabetic nephropathy (see Table 1).

2. Clinical presentation on admission

**Table 1:** Summary of patients’ demographics, underlying diseases, clinical data, blood results, and hemodialysis details

Patient	P1	P2	P3	P4
Sex	Male	Female	Female	Male
Age (years)	31	65	69	61
Underlying disease	IgA nephropathy	Chronic glomerulonephritis	Diabetic nephropathy	Diabetic nephropathy
Comorbid disease	No	No	Systemic scleroderma coronary heart disease	coronary heart disease
Digestive system diseases	No	Chronic superficial gastritis, constipation	Constipation	Constipation
Long-term oral drug administration	No	No	Glucocorticoids	Aspirin
Frequency of HD	12 h/week	8 h/week	10 h/week	12 h/week
Frequency of HDF	8 h/month	No	4 h/month	4 h/month
Years on HD	3	2	3	1
Anticoagulants and doses used in dialysis	Low-molecular-weight heparin 3000 U/time	Low-molecular-weight heparin 1000 U/time	Low-molecular-weight heparin 3500 U/time	Low-molecular-weight heparin 5000 U/time
Vascular access	Arterio-venous fistula	Right femoral vein catheterization (heparin saline 1:1 tube sealing)	Semi-permanent catheterization of right internal jugular vein (sealed with heparin only)	Arterio-venous fistula
Accompanied by cardiac insufficiency	No	No	Yes	Yes
Accompanied by thrombocytopenia	No (123*10^12^/L)	No (256*10^12^/L)	Yes (78*10^12^/L)	No (201*10^12^/L)
With coagulation dysfunction	No (PT 13.1 s)	Yes (PT 19.3 s)	No (PT 13.8 s)	Yes (PT 18.1 s)
Serum creatinine level before dialysis (μmol/L)^a^	1000	800	500	800
BUN (mmol/L)	15.4	16.78	17.2	15.3
Pre-onset Hb^b^ levels (g/L)	115	75	107	107
Hb level at onset (g/L)	87	43	85	99
Kt/V	1.44	1.25	1.13	1.32

^a^Values are approximate

^b^Hemoglobin

P1: After eating, P1 experienced severe vomiting 30 min before dialysis. The vomit contained gastric contents. Hematemesis occurred two hours after the start of dialysis and was bright red with a volume of approximately 200 mL. The patient complained of chest and back discomfort.

P2: P2 experienced vomiting one day before admission. There was no obvious cause for the vomiting, which was dark red. The patient also had nausea and fatigue.

P3: P3 experienced nausea and vomiting one day prior to admission, and the vomit contained gastric contents. This was accompanied by abdominal pain, diarrhea, watery stools, and fever. The highest body temperature recorded for P3 was 38.2 ℃.

P4: One week before admission, P4 developed chest tightness and shortness of breath accompanied by nausea and vomiting. The vomit was black and occurred on three occasions.

**Fig. 1:**
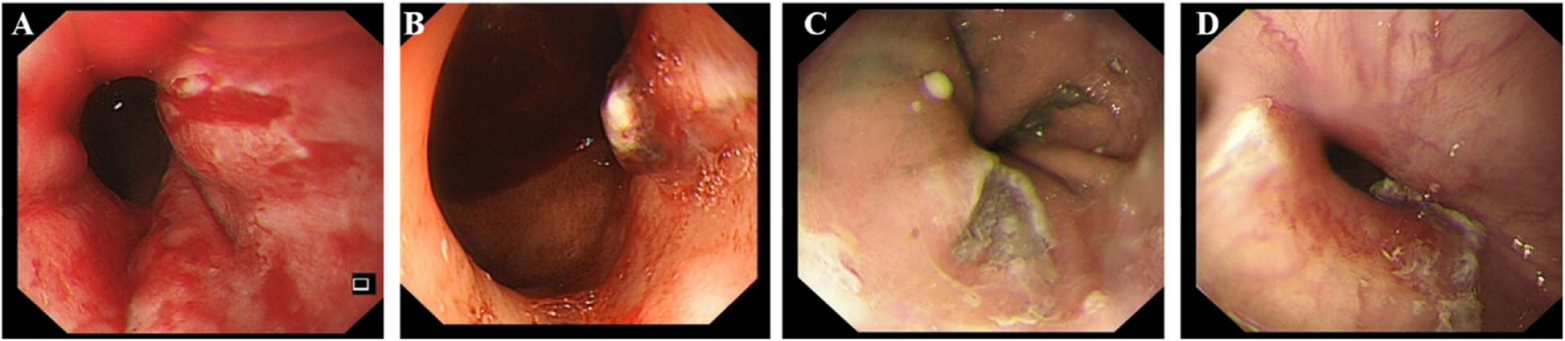
Gastroscopy results for four patients. **A** The gastroscopy for P1 revealed a MWS. **B** The gastroscopy for P2 revealed MWS with thrombosis. **C** The gastroscopy for P3 revealed a tear in the cardiac mucosa that may have been accompanied by bleeding. **D** The gastroscopy for P4 revealed flaky erosions at the entrance of the cardia and that tearing might be possible

3. Gastroscopy results (Fig. 1)

P1: The gastroscopy for P1 revealed a cardia submucosal hematoma.

P2: The first gastroscopy performed for P2 after admission showed cardia mucosal hemorrhage and a poor treatment effectiveness. A second gastroscopy revealed MWS with thrombosis.

P3: The gastroscopy for P3 revealed a tear in the cardia mucosa that might have been accompanied by bleeding.

P4: The gastroscopy revealed flaky erosions at the entrance of the cardia and possible tearing.

4. Treatment

After admission, the four patients were given symptomatic and supportive treatments that included fasting, acid suppression, gastric mucosal protection, hemostasis, fluid replacement, blood transfusion (to correct anemia), and heparin-free dialysis.

**Fig. 2:**
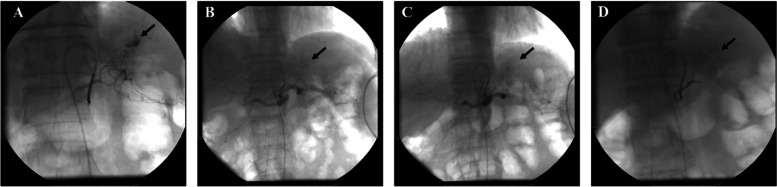
Left gastric artery hemorrhage embolism diagram for patient 2. **A** Celiac trunk arteriography showing bleeding from the left gastric artery (black arrow). **B** Super selective left gastric angiography showing bleeding (black arrow). **C** Super selective left gastric artery embolization showing the cessation of bleeding (black arrow). **D** Celiac artery angiography showing the left gastric artery embolism (black arrow)

**Fig. 3:**
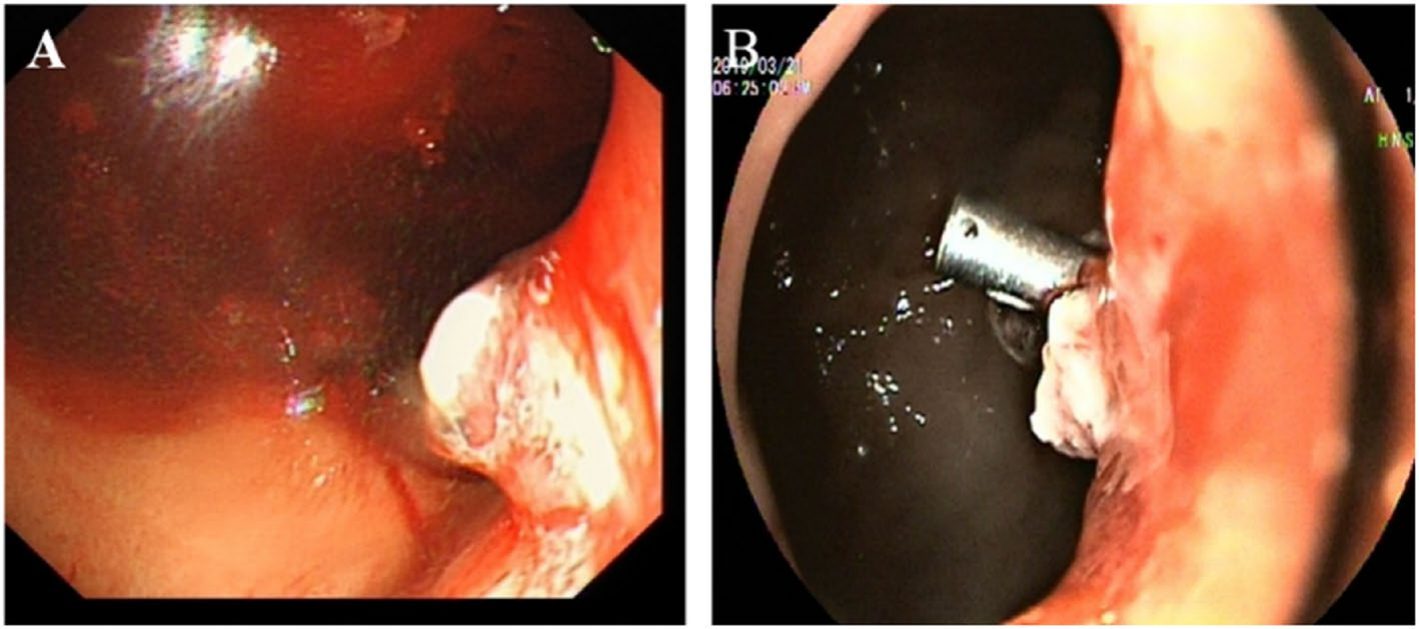
Gastroscopic hemostasis in patient 2. **A** Tear and bleeding of the cardia mucosa. **B** Titanium clip to stop the bleeding

P2: The patient first underwent an interventional lower gastric artery embolization due to the large amount of bleeding after admission. The interventional effect was average. Since the patient still had bleeding, three gastroscopic titanium clamps were used to stop the bleeding (Figs. 2 and 3).

P2, P3, and P4: Due to the poor coagulation function, coagulation factors and plasma were administered to stop the bleeding.

P2 and P3: A 10% sodium chloride solution was used for central venous catheter sealing.

5. Prognosis

P3: P3 did not bleed or vomit blood by the second day after admission. On the 6th day, the stool color turned yellow, and the fecal occult blood test was negative. P3 resumed eating on the 9th day. On the 12th day, the patient's blood pressure decreased to 84/56 mmHg, and the heart rate was 100 beats/min, with a uniform sinus rhythm. After retesting results showed hemoglobin and blood potassium values of 93 g/L and 4.1 mmol/L, respectively. P3 stopped treatment and died outside the hospital.

P1, P2, and P4: The remaining three patients recovered and were discharged after treatment. Routine stool examinations were carried out one month after the discharge, and the results were normal. No obvious gastrointestinal bleeding was present in the three patients.

## Discussion and conclusions

MWS refers to non-transmural lacerations at the gastroesophageal junction that are generally caused by severe vomiting but can result from other causes, including increased intra-abdominal pressure and a rapid increase in the intragastric pressure (i.e., vomiting, severe coughing, epilepsy, acute or severe asthma, constipation, or endoscopy). It was first reported by Mallory and Weiss in 1929 [2] and accounts for 1% to 15% of the causes of upper gastrointestinal bleeding in adults [3]. In most cases, MWS is a self-limiting, benign disease [4, 5]. Mild cases may be asymptomatic. In 85% of cases, the main symptom is hematemesis. The quantity of blood is variable. The diagnosis is based on gastroscopy, where a tear of the mucosa, submucosal hematoma, and bleeding are the diagnostic criteria. The diagnosis and cause of MWS are usually clear.

HD patients tend to have gastrointestinal bleeding, and among them, the proportion with MWS is quite different. A study in the United States in 2015 found that the proportion of MWS in HD patients with upper gastrointestinal bleeding was 2.34% [6], and a study in China examined 68 patients with upper gastrointestinal bleeding, of which MWS accounted for 11.8%. The reason for the large gap in the proportion of MWS reported in the literature is that there may be mild symptoms, which are not identified.

The four patients with HD in this article had a history of vomiting with varying degrees of severity. However, three of them reported a history of mild vomiting, yet they developed MWS. The analysis of clinical data of these patients reveled that they had certain risk factors for MWS. According to the literature, age, uremic toxins [7], anemia, certain drugs [8] (i.e., nonsteroidal anti-inflammatory drugs), vascular calcification, cardia insufficiency, related diseases such as diabetes, and various types of vasculitis are predisposing factors for MWS. Age is a factor because the strength of collagen fibers in the submucosa of the esophagus and cardia tend to gradually weaken and can easily be torn by external forces [9]. Various uremic toxins can cause intestinal motility disorders, which can cause the patient to be more prone to bleeding and vomiting [10–13]. If the mucosa has been in a state of damage, congestion, and prolonged edema, the hemodynamic changes that occur during HD aggravate the intermittent ischemic state of the intestinal mucosa, leading to further aggravation and mucosal damage. Therefore, HD patients are more prone to MWS. Among the four patients in our study, two were middle-aged (61 and 65 years old), and one was an elderly female (69 years old). These three patients had a history of constipation, and they had received dialysis for > 1 year. Their average blood creatinine levels before dialysis reached 700 μmol/L. All patients had varying degrees of anemia, and one of them was moderately anemic. Two patients had a history of diabetes, one with a long-term use of oral hormones and the other one with aspirin. These factors most likely caused these three patients to develop MWS without severe vomiting.

The diagnosis of MWS in these clinical cases suggests that a delayed or completely missed diagnosis would not be uncommon. The main clinical symptom of MWS is upper gastrointestinal bleeding, which can present as hematemesis and melena, depending on the amount of bleeding. However, for HD patients, mild vomiting (or other reasons, as stated above) can lead to MWS. In this case study, one patient had a large amount of short-term bleeding, manifesting as obvious hematemesis. The patient vomited violently, so the diagnosis and treatment were carried out as soon as possible. However, the symptoms of hematemesis in the other three patients were not obvious and were possibly masked by other symptoms, such as chest tightness, shortness of breath, diarrhea, and fever. At the same time, due to the presence of constipation, examining the early stage of melena was impossible, and the first diagnosis was incorrect. Such cases serve as a warning. For those with a history of vomiting but no obvious bleeding symptoms, close observation is necessary. Without an obvious history of hypercoagulability, local citrate anticoagulation can be considered for the first HD after vomiting. In addition, you should observe the occult blood in the stool, and complete gastroscopy, if necessary. In addition, some conditions that can lead to MWS, such as severe cough, epilepsy, acute severe asthma, and constipation, can occur in HD patients. When these symptoms occur, we also need to closely observe the bleeding of patients and make a timely diagnosis.

The current treatment of MWS is based on gastrointestinal bleeding and tearing. Conservative treatment approaches include acid suppression and hemostasis, and if necessary, hemostasis using gastroscopy and interventional vascular embolization. Since MWS is mostly self-limited and recurrence is uncommon, a conservative approach would be appropriate for most patients. However, for HD patients with MWS, if the amount of bleeding is large, long-term conservative treatment will aggravate the disease and treatment becomes more difficult. These other factors include an impaired platelet function and a decreased platelet count [14, 15]. Uremic toxins, such as urea, phenol, and guanidinosuccinic acid, are also closely related to platelet dysfunction and should be monitored [16]. In addition, the regular use of anticoagulants in long-term HD patients, as well as the effects of their toxins on the bone marrow, can cause various coagulation disorders [17]. Furthermore, HD patients often have other complications such as hypertension, diabetes, and coronary heart disease. These comorbidities complicate the overall disease [18, 19]. Finally, most HD patients have an abnormal calcium and phosphorus metabolism, calcification of blood vessels throughout the body, and decreased vascular elasticity [20], making them more prone to bleeding, and the bleeding is not easily stopped in such cases. In this case study, three patients were treated using a conservative approach, and the results were acceptable, but the required treatment time was longer. One patient died of heart failure, and another patient had severe bleeding. Hemostasis with a titanium clip during gastroscopy and a single interventional vascular embolization were performed in another patient. The reason for the poor effect of the repeated surgery in this patient was poor coagulation and severe local bleeding. In addition to non-heparin or local citrate anticoagulation dialysis after a timely diagnosis, sealing the central venous catheters without heparin or with citrate is also a very important issue that is easily overlooked. Therefore, when treating HD patients with MWS, conservative treatment is not recommended, and stopping the bleeding using a gastroscope or interventional therapy, or even surgical treatment, is recommended. At the same time, paying attention to the coagulation function during treatment is very necessary.

Based on multiple database searches using different retrieval methods, case reports of patients with regular HD combined with MWS are relatively rare. Only five cases of chronic kidney disease combined with MWS have been reported in Japan, and little data are available from these cases. However, according to the diagnosis and treatment summary of four patients in this study, we believe that the clinical manifestations of mild MWS are easily hidden, which leads to delayed diagnosis. In order to ensure safe treatment, we should pay attention to the existence of coagulation function and various complications. For patients with severe bleeding, endoscopic hemostasis is the first choice; Interventional methods can be provided if necessary, but necessary drug treatment is also crucial.

## Data Availability

All data related to this case report are within the manuscript.

## Abbreviations

HD: Hemodialysis
MWS: Mallory-Weiss syndrome
